# Effects of habitual endurance and resistance exercise on insulin action in primary human skeletal muscle stem cells

**DOI:** 10.14814/phy2.70600

**Published:** 2025-09-30

**Authors:** Polina Krassovskaia, Filip Jevtovic, Donghai Zheng, John Noone, Reichelle X. Yeo, Maria F. Pino, Cynthia L. Stowe, Shannon S. Emilson, Nicolas Musi, Kim M. Huffman, Caitlin Hebert, Sophia Bowen, Simona Zarini, Eric Ravussin, Nicholas T. Broskey, William E. Krauss, Bryan C. Bergman, Lauren Sparks, Joseph A. Houmard

**Affiliations:** ^1^ Department of Cancer Biology Wake Forest University Winston Salem North Carolina USA; ^2^ Department of Kinesiology East Carolina University Greenville North Carolina USA; ^3^ Human Performance Laboratory East Carolina University Greenville North Carolina USA; ^4^ East Carolina Diabetes and Obesity Institute East Carolina University Greenville North Carolina USA; ^5^ AdventHealth Translational Research Institute Orlando Florida USA; ^6^ Department of Physical Education and Sport Sciences, Faculty of Education and Health Sciences University of Limerick Limerick Ireland; ^7^ Department of Biostatistics and Data Science Wake Forest University School of Medicine Winston‐Salem North Carolina USA; ^8^ Department of Medicine Cedars‐Sinai Medical Center Los Angeles California USA; ^9^ Duke University School of Medicine Durham North Carolina USA; ^10^ Pennington Biomedical Research Center Baton Rouge Louisiana USA; ^11^ Anschutz Medical Campus University of Colorado Aurora Colorado USA

**Keywords:** exercise, insulin action, insulin signaling, primary myotubes, skeletal muscle

## Abstract

Endurance‐oriented exercise typically enhances insulin action in skeletal muscle; however, relatively little is known about the impact of resistance exercise. In the present study, insulin action was determined in primary human skeletal muscle stem cells (HSkMCs) isolated from habitual endurance and resistance exercisers and sedentary controls (*N* = 8–9/group). Insulin action was assessed by insulin‐stimulated glycogen synthesis and glucose oxidation using 14C‐labeled glucose and insulin signal transduction measured as phosphorylation of Akt (Ser^473^) and AS160 (Thr^640^). No differences were detected in basal and insulin‐stimulated glycogen synthesis, glucose oxidation, and insulin signal transduction between the endurance and resistance exercisers. When HSkMCs were challenged by a fatty‐acid treatment which induced insulin resistance, no differential protection was detected with either exercise training modality. When data from the habitual endurance and resistance exercise groups were combined (EX) and compared to sedentary controls, HSkMC from EX exhibited greater rates of insulin‐stimulated glycogen synthesis. However, Akt and AS160 phosphorylation were similar between EX and sedentary individuals. Exercise training provided no protection against fatty‐acid‐induced insulin resistance across any measure of insulin action. These data suggest that habitual exercise, including resistance training, improves insulin action in skeletal muscle but may not offer intrinsic protection against fatty‐acid‐induced insulin resistance.

## 1 INTRODUCTION

One of the most prominent changes observed with exercise is an improvement in insulin sensitivity in skeletal muscle and the whole‐body levels (Reaven & Chen, 1988). While most health organizations recommend regular moderate‐intensity aerobic exercise to improve insulin action, a gap in knowledge remains regarding the effectiveness of different exercise modalities such as resistance exercise. For example, in the STRRIDE AT/RT (Studies Targeting Risk Reduction Interventions through Defined Exercise‐Aerobic Training and/or Resistance Training) studies, fasting insulin, HOMA‐IR, and the acute insulin response to intravenous glucose were significantly lower after 8 months of aerobic (EE) but not resistance (RE) training in sedentary adults with dyslipidemia (AbouAssi et al., 2015). This is contrary to the DARE (Diabetes Aerobic and Resistance Exercise) study where patients with type 2 diabetes had a significant reduction in hemoglobin A_1C_ with both exercise modalities (Sigal et al., 2007) and the Health Benefits of Aerobic and Resistance Training in Individuals with Type 2 Diabetes (HART‐D) study where there was no hemoglobin A_1C_ improvement with either exercise mode (Church et al., 2010). However, studies utilizing the hyperinsulinemic‐euglycemic clamp have reported improvements in insulin action with RE training in participants with and without type 2 diabetes (Holten et al., 2004; Ishii et al., 1998). Such differences in physiological responses to exercise can be attributed to intrinsic factors (e.g., sex, age, disease states), variations in the exercise prescriptions (e.g., intensity, duration, volume), and the specific aspect of insulin action measured (Noone et al., 2024) (e.g., HOMA‐IR, glucose disposal rate, Hb A_1C_). Accordingly, a more direct assessment of glucose metabolism in human skeletal muscle is necessary to discern if there are differences between EE and RE training.

Primary human skeletal muscle stem cells (HSkMCs) provide a model for directly studying insulin action in human skeletal muscle and reside between the sarcolemma and basal lamina of the muscle fiber (Yeo et al., 2024). With the contractile stimulus, HSkMCs become activated, proliferate, and either fuse to form new muscle fibers or fuse to existing fibers and contribute their myonuclei, transferring (epi)genetic information to existing muscle fibers and daughter satellite cells (Ceccarelli et al., 2017; Snijders et al., 2015). As satellite cells respond to the muscle microenvironment, it is reasonable to postulate that they are affected differently by EE and RE training. For example, while studies have reported improvements in insulin action in HSkMCs isolated from individuals performing 8–12 weeks of EE (or combined EE and RE), it is not clear if there are any differences between EE and RE training when each is performed alone (Bourlier et al., 2013; Lund et al., 2017). Some evidence for differential mode effects comes from fetal stem cells and stem cells undergoing myogenesis where RE increased glucose oxidation more than EE, while both modes of exercise training enhanced insulin action (Jevtovic, Zheng, Houmard, Kern, et al., 2023; Jevtovic, Zheng, Houmard, Krassovskaia, et al., 2023). Another potential benefit to be gained with exercise training is a protective effect against metabolic insults that induce insulin resistance, such as lipid exposure. Such protection has been observed in rodent models where both RE (Kim et al., 2022) and EE (Morris et al., 2019) training alleviate the insulin resistance induced by a high‐fat diet, but these findings have yet to be confirmed in humans.

The purpose of the present study was to determine the effects of EE and RE training on insulin action in human skeletal muscle. Insulin‐mediated glycogen synthesis, glucose oxidation, and insulin signal transduction were measured in HSkMCs from EE and RE trained participants in the Molecular Transducers of Physical Activity Consortium (MoTrPAC) study (MoTrPAC Study Group et al., 2024; Sanford et al., 2020). In addition, HSkMCs were exposed to a lipid treatment which induced insulin resistance (Bikman et al., 2010) to determine if a protective effect of exercise training was conferred. We hypothesized that habitual EE and RE training would result in differential effects on HSkMC insulin‐stimulated glucose metabolism and insulin signaling, and that prior training status would confer protection against fatty‐acid induced insulin resistance.

## 2 MATERIALS AND METHODS

### 2.1 Design and participants

Participants were part of a study titled “Investigating the effects of aerobic and resistance training in vivo on skeletal muscle metabolism in vitro in primary human cells,” (MoTrMyo), which was an ancillary study to the “Molecular Transducers of Physical Activity Consortium” (MoTrPAC) parent study (Sanford et al., 2020). Briefly, a highly active endurance exercise group (EE), a highly active resistance exercise group (RE), and a sedentary group (SED) were recruited for MoTrPAC after providing written and verbal informed consent for an additional muscle biopsy (*vastus lateralis*) to be obtained in the resting condition after an overnight fast. EE were defined as participating in a mode of EE (running, brisk or power walking, cycling, elliptical, etc.) for ≥240 min/week for ≥1 year that resulted in increased heart rate, rapid breathing, and sweating. The EE must have included cycling at least 2 days/week for at least 120 min per week. If RE was included in the previous year, such training must have been limited to ≤2 days/week of upper body exercise involving ≤2 muscle groups and ≤1 day/week of lower body exercise.

RE were defined as individuals participating in RE training sessions of ≥3 upper and ≥3 lower body muscle groups on ≥2 times/week for a period ≥1 year. The RE was at an intensity and dose sufficient to increase strength and muscle mass. If EE training was included in the previous year, such training was limited to ≤90 min/week of vigorous exercise, with no limit on the number of cycling days per week.

A sedentary participant was defined as self‐reporting a consistent pattern of minimal exercise in the previous year of no more than 1 day per week, lasting no more than 60 min of regular EE (e.g., brisk walking, jogging, running, cycling, elliptical, or swimming activity that resulted in feelings of increased heart rate, rapid breathing, and/or sweating) or RE (resulting in muscular fatigue) of no more than 1 day per week. Within these criteria, persons cycling as a mode of transportation to and from work ≥1 day/week were not considered sedentary.

Inclusion/exclusion criteria were those of the parent study (MoTrPAC) and are presented in more detail elsewhere (MoTrPAC Study Group et al., 2024). Briefly, exclusion criteria included major health conditions or use of medications where exercise could cause additional complications, participant safety concerns, or influenced the molecular responses or phenotypic changes to exercise.

### 2.2 Analytical approach

Compared to the parent study, the total number of participants in MoTrMyo was smaller, predominantly male, and consisted of a wide range of cardiorespiratory endurance (VO_2peak_) and strength (maximal leg extension) as well as age. The data presented are from a subset of MoTrMyo RE and EE participants who were individually matched across groups for age, gender, and race to minimize variation induced by these parameters. To maximize the effects of exercise training, the habitually trained individuals selected were in the upper tertiles for VO_2peak_ (>35 mL/kg/min; EE) and leg extension strength (>250 nm; RE). There were no statistical adjustments for age or sex.

### 2.3 Phenotyping procedures

The parent study (MoTrPAC) utilized screening/phenotyping procedures described in detail elsewhere (MoTrPAC Study Group et al., 2024). For the present study, data from indices indicative of exercise training status (i.e., muscle strength represented as isometric knee average peak torque and cardiorespiratory fitness represented as VO_2_peak) and anthropometrics were utilized. Procedures were standardized across the participating sites (MoTrPAC Study Group et al., 2024).

Quadriceps muscle strength was determined with isometric knee extension of the dominant leg using a dynamometer. A cardiopulmonary exercise test (CPET) on a cycle ergometer was used to assess cardiorespiratory fitness and determine peak oxygen consumption (VO_2peak_). The cycle ergometer (Lode Excalibur) and leg positioning were standardized across sites. Expired gases were measured by indirect calorimetry using a metabolic cart calibrated prior to each test. To accommodate the anticipated variability in cardiorespiratory fitness of the study participants, an appropriate ramping protocol was selected as described in Jakicic et al (Study Group et al., 2024). Peak exercise was deemed to occur if the respiratory exchange ratio (RER) was ≥1.05. VO_2peak_ was represented as the average of the final two 30‐s intervals. Physical activity was measured using self‐report and information obtained on participation in the types and amounts of leisure‐time/recreational, occupational, household, transportation, recreational, and sedentary activity.

### 2.4 Skeletal muscle cell culture

As described elsewhere (Bikman et al., 2010; MoTrPAC Study Group et al., 2024; Park et al., 2019) a portion of the skeletal muscle biopsy (~50 mg) was minced and digested for 30 min in a 0.25% trypsin (15090‐046; Gibco)/0.068% collagenase type IV (17104‐019; Gibco) solution supplemented with 0.05% EDTA and 0.1% bovine serum albumin (BSA) (A8412; Sigma) at 37°C in a shaking water bath. Digested muscle was pre‐plated in a 60 mm dish for 1 h at 37°C with 5% CO_2_ for removal of fibroblasts, and then transferred into a collagen‐coated T‐25 (430641; Corning) in 16% growth media (low‐glucose DMEM), 16% FBS (16140‐071; Gibco), 0.05% BSA, 0.1% 50 mg/mL Gentamicin (15750‐060; Gibco), 0.1% 1uM Dexamethasone (D8893; Sigma), 0.1% human epithelial growth factor (EGF) (13247‐051; Invitrogen), 0.02% 250 μg/mL Amphotericin B (15290‐018; Gibco)). After 70% confluency, cells were expanded in non‐collagen coated T‐75 cell culture flasks (431464; Corning) in 10% growth media (low‐glucose DMEM, 10% FBS, 0.05% BSA, 0.1% 50 mg/mL Gentamicin, 0.1% 1uM Dexamethasone, 0.1% human EGF, 0.02% 250 μg/mL Amphotericin B). At 70% confluency these expanded cells were immunopurified by magnetic cell separation using surface marker antiCD56 (130‐050‐401; Milteny Biotec). Each 10^6^ cells were combined with 20 μL of CD56 Ab‐Microbeads diluted in 80 μL MACS buffer (PBS supplemented with 0.05% BSA and 2 mM EDTA (15575‐038; Invitrogen)) and incubated at 4°C for 15 min before washing in 1 mL MACS buffer (×2) and centrifugation at 350*g* x 10 min (×2). Cells were then separated using MACS columns (130‐042‐201; Milteny Biotec), and CD56+ cells were plated in non‐collagen coated T‐75 cell culture flasks in 10% growth media. At 70% confluency, cells were split to 12‐well CellBIND plates (3336; Corning) and switched to differentiation media (low‐glucose DMEM, 2% FBS, 0.2% BSA, 2% penicillin/streptomycin (15070063; ThermoFisher) at 90% confluency. All experiments were done on Day 6 of differentiation between passages 4 and 5. As multiple sites were involved, an initial training session was convened in a single location to ensure uniform techniques.

### 2.5 Palmitate treatment

Palmitic acid (P5585; Sigma) dissolved in ethanol (200 mM) was added into serum‐starvation media (low‐glucose DMEM with 1% BSA) to a final concentration of 450 μM. This cocktail was incubated at 37°C in a shaking water bath for at least 1 h. Initial dose–response experiments were conducted in myotubes and indicated that 20 h, 450 μM palmitate incubation decreased insulin‐stimulated glycogen synthesis (data not shown). Moreover, this concentration has been previously shown to induce insulin resistance in myotubes (Bikman et al., 2010). Serum‐starvation media with or without palmitate was started on Day 5 of differentiation for 20 h. The palmitate treatment is abbreviated as 20hFA.

### 2.6 Glucose oxidation

Myotubes were washed with DPBS and incubated with media containing d‐[1‐^14^C] glucose (NEC042V250UC; Perkin‐Elmer; 1.5 μCi/mL, 5.0 mM glucose) for 2 h at 37°C, as described elsewhere (Hinkley et al., 2017; Park et al., 2019; Zou et al., 2019). Following incubation, experimental media was transferred into a customized 48‐well trapping plate with fabricated grooves between two continuous wells. CO_2_ in the media was acid trapped in 1N NaOH (SS255; ThermoFisher) with the addition of 70% perchloric acid (A2296; ThermoFisher). Liquid scintillation counting (Revvity Tri‐Carb 4810 TR) was used to measure the incorporation of ^14^C‐glucose into CO_2_ using the conditioned NaOH to derive a rate of complete oxidation. Myotubes were then washed with DPBS and solubilized using 0.5% SDS (BP2436200; ThermoFisher). Protein content of lysates was measured using a BCA assay (bicinchoninic acid assay) (23225; Pierce Biotechnology, Inc.), and all rates of oxidation were normalized to protein content and internal controls.

### 2.7 Glycogen synthesis

Insulin‐stimulated glycogen synthesis was determined as an index of insulin sensitivity as previously described (Bikman et al., 2010; Park et al., 2019; Park et al., 2024; Zou et al., 2019). Myotubes underwent 3 h of serum starvation, 2‐h incubation with ^14^C‐glucose, two PBS washes, and lysing with 0.5% SDS. An aliquot of the lysate was combined with carrier glycogen (1 mg) (G0885; Sigma) and denatured at 100°C for 1 h. Ice‐cold ethanol was added to the denatured lysates, and samples were spun overnight at 4°C for glycogen precipitation. The next day, glycogen pellets were centrifuged at 11,100*g* for 15 min at 4°C, washed with 70% ethanol, and centrifuged again. The glycogen pellets were then resuspended in dH_2_O, and the incorporation of ^14^C‐glucose into glycogen was determined with liquid scintillation counting.

### 2.8 Immunoblotting

Myotubes were incubated in the presence or absence of 100 nM of insulin for 10 min, then rinsed with DPBS and lysed in ice‐cold mammalian protein extraction reagent (M‐PER) (78501; ThermoFisher) containing phosphatase 2 and 3 (P5726; Sigma) (P0044; Sigma) and protease inhibitor cocktails (P8340; Sigma), along with 10 mM sodium orthovanadate (450243; Sigma). Samples were sonicated for 5 s, then rotated end‐over‐end at 4°C for 2 h and centrifuged at 12,000×*g* at 4°C for 15 min. The supernatant was collected, and protein concentrations were determined (BCA Assay). Cell lysates were mixed with 4× Laemmli Sample Buffer (1610747; Bio‐Rad), separated by SDS‐PAGE electrophoresis, and transferred to the nitrocellulose membrane using the Trans‐Blot® TurboTM Transfer System (Bio‐Rad). Membranes were blocked with 5% BSA in TBS‐T (1× TBS, 0.1% Tween‐20) for 1 h, then incubated overnight at 4°C with primary antibodies against Akt (total)(Cell Signaling, 9272), Akt (Ser473) (Cell Signaling, 9271), AS160 (total) (Cell Signaling, 2670), AS160 (Thr642) (Cell Signaling, 4288), and β‐Actin (Cell Signaling, 3700) diluted in 5% BSA in TBS‐T. Following primary incubation, membranes were washed with TBS‐T (1× TBS, 0.1% Tween‐20) and incubated with fluorophore‐conjugated secondary antibody at a 1:10,000 dilution for 1 h. Secondary antibodies (926‐32211, 926‐32210, 926‐68072; LI‐COR) were diluted in 5% BSA in TBS‐T (1× TBS, 0.1% Tween‐20). Membranes were washed with TBS‐T and scanned on an Odyssey near‐infrared imager (Li‐Cor). Images were quantified on Image Lab Software for PC Version 6.1 (Bio‐Rd). All data were normalized to β‐Actin protein expression.

### 2.9 Statistical analyses

The primary goal of this study was to determine if training modality (EE or RE) differentially affected insulin action; the initial comparison was thus between the EE and RE groups with a subsequent comparison of exercise trained to the SED participants. The EE and RE groups were matched for age, gender, and race and were in the upper tertile of the study sample (MoTrMyo) for VO_2peak_ and isometric strength. All data collected in [^14^C]‐glucose experiments was performed in technical triplicate, and the mean of the three replicates was used as the representative value for each participant and in statistical analyses. Unpaired or paired two‐tailed *t*‐test or two‐ or three‐way analysis of variance (ANOVA) with Bonferroni correction were used to determine statistical significance. Factors were group (EE vs. RE; SED vs. EX), insulin, and condition (standard vs. 20hFA). Data for insulin action were also expressed as fold changes (insulin stimulated divided by basal). Relationships were determined with Pearson correlation coefficients. Statistical significance was set as *p* ≤ 0.05. All data were expressed as mean ± SEM.

## 3 RESULTS

### 3.1 EE and RE participant characteristics

Participant characteristics are reported in Table 1. A difference was not detected between the EE and RE participants in age or minutes of exercise per week from self‐report. EE had a higher VO_2peak_ (*p* = 0.0002) and RE had a higher isometric knee torque (*p* < 0.0001) and BMI (*p* = 0.0274). Both exercise groups were comparable in terms of sex and race distribution.

**TABLE 1:** Participant characteristics of highly‐active endurance (EE) and resistance (RE) exercisers.

	EE, *N* = 8	RE, *N* = 8	*p*‐value
Age (years)	38.1 ± 7.3	31.5 ± 7.6	0.0823
Sex	4 F/4 M	3 F/5 M	
Race	7 C/1 H	5 C/2 AA/1 AS	
BMI (kg/m^2^)	22.3 ± 2.4	27.0 ± 1.6	0.0274
Minutes of exercise/week	406.3 ± 111.3	352.5 ± 227.8	0.0615
VO_2_peak (mL/kg/min)	49.0 ± 10.6	31.7 ± 5.6	0.0002
Isometric Knee Avg Peak Torque (Nm)	172.5 ± 40.7	313.6 ± 73.9	<0.0001

*Note*: Data are presented as mean ± SD.

Abbreviations: AA, African American; AS, Asian; C, Caucasian; F, female; H, Hispanic; M, male.

### 3.2 Glycogen synthesis and glucose oxidation for EE and RE

**FIGURE 1:**
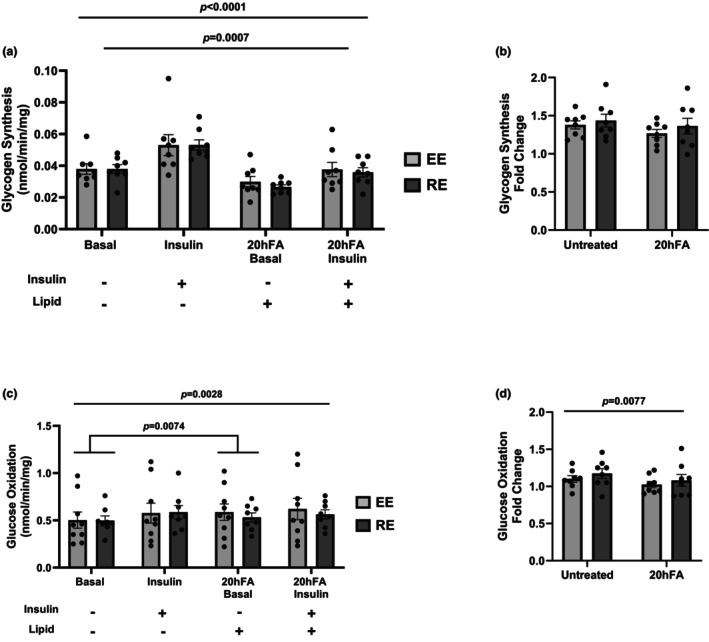
Glycogen synthesis or glucose oxidation rates in highly active endurance (EE) and resistance (RE) exercisers. (a) Absolute rates of glycogen synthesis in both basal and insulin‐stimulated standard and palmitate treated (20hFA) conditions. (b) Relative (fold change over basal) insulin‐stimulated glycogen synthesis rates in the standard and 20hFA conditions. (c) Absolute rates of glucose oxidation in both basal and insulin‐stimulated standard and 20hFA conditions. (d) Relative (fold change over basal) insulin‐stimulated glucose oxidation in the standard and 20hFA conditions. A three‐way ANOVA was used to detect significance between groups, insulin, and/or treatment, and a two‐tailed *t*‐test to distinguish meaningful differences. There was a main effect of insulin (*p* < 0.0001) and 20hFA (*p* = 0.0007) for glycogen synthesis. There was a main effect of insulin for glucose oxidation (*p* = 0.0028), and there was a main effect of 20hFA treatment in the basal state only (*p* = 0.0074). There was a main effect of 20hFA treatment for glucose oxidation fold change (*p* = 0.0077). There was no group effect for either glycogen synthesis or glucose oxidation (*p* > 0.05). Data are presented as mean ± SEM. *N* = 8 per group. EE, highly active endurance exercise group; RE, highly active resistance exercise group; 20hFA, 20‐h 450 μM palmitate treatment.

Insulin increased glycogen synthesis in EE and RE in both the standard and 20hFA conditions (*p* < 0.001) (Figure 1a). Additionally, there was a main effect of 20hFA treatment evident as blunted basal and insulin‐stimulated glycogen synthesis rates in both EE and RE (*p* = 0.0007) (Figure 1a). No difference in insulin‐stimulated glycogen synthesis was detected between EE and RE with (0.0376 ± 0.013 vs. 0.0359 ± 0.0083 nmol/min/mg, respectively; *p* > 0.05) or without (0.0530 ± 0.018 vs. 0.0531 ± 0.0093 nmol/min/mg; *p* > 0.05) 20hFA treatment (Figure 1a). Though 20hFA treatment blunted absolute rates of glycogen synthesis, no difference was detected in the relative change (fold‐change) in insulin‐stimulated glycogen synthesis between RE and EE in either the standard or 20hFA conditions (Figure 1b), indicating high‐dose palmitate treatment blunts overall glycogen synthesis in absolute terms but does not alter sensitivity to insulin. Insulin stimulation increased glucose oxidation in the standard (*p* = 0.0028) but not in the 20hFA condition (Figure 1c). A difference was not detected in the relative change in glucose oxidation with insulin between EE and RE in either the standard or 20hFA conditions (Figure 1d).

### 3.3 Insulin signal transduction for EE and RE

**FIGURE 2:**
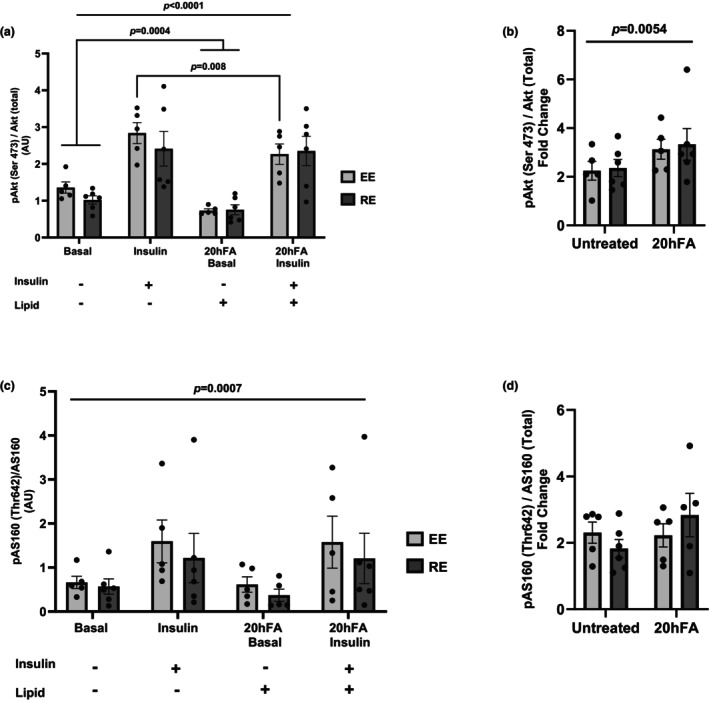
Insulin signal transduction in the absence or presence of 100 nM insulin and/or 20 h of lipid incubation. (a) Akt (Ser^473^) phosphorylation. (b) Relative (fold change over basal) Akt (Ser^473^) phosphorylation. (c) AS160 (Thr^642^) phosphorylation. (d) Relative (fold change over basal) AS160 (Thr^642^) phosphorylation. A three‐way ANOVA was used to detect significance between groups, insulin, and/or treatment, and a two‐tailed *t*‐test was used to distinguish meaningful differences. There was a main effect of insulin for both Akt (*p* < 0.001) and AS160 (*p* = 0.0007). There was a main effect of 20hFA treatment in the basal, but not insulin, state for Akt (*p* = 0.0004) and in Akt fold change (*p* = 0.0054). In the insulin‐stimulated condition, 20hFA treatment resulted in decreased Akt phosphorylation for EE only (*p* = 0.008). There was no group effect for either Akt or AS160 (*p* > 0.05). Data are presented as mean ± SEM. *N* = 5 for EE, *n* = 6 for RE. EE, highly active endurance exercise group; RE, highly active resistance exercise group; 20hFA, 20‐h 450 μM palmitate treatment.

No differences between EE and RE were detected in either Akt (Ser^473^) or AS160 (Thr^642^) phosphorylation under standard or 20hFA conditions (Figure 2a,c). There was a significant main effect with insulin for both Akt (*p* < 0.0001) and AS160 (*p* = 0.0007) (Figure 2a,c), and the 20hFA treatment had a main effect only on basal Akt phosphorylation (*p* = 0.0004). For only EE, insulin‐stimulated Akt phosphorylation was lowered with 20hFA treatment compared to the standard state (*p* = 0.008).

### 3.4 SED and EX participant characteristics

As differences were not detected between EE and RE, the data were combined into an exercise (EX) group to compare outcomes of habitual exercisers to sedentary (SED) participants. Participant characteristics of EX and SED participants are reported in Table 2. No differences between SED and EX were detected in age or isometric knee torque. SED had a higher BMI compared to EX (*p* = 0.0103) and lower VO_2peak_ (*p* < 0.0001).

**TABLE 2:** Subject characteristics of sedentary individuals (SED) and highly active exercisers (EX).

	SED, *N* = 9	EX, *N* = 16	*p*‐value
Age (years)	38.1 ± 9.2	34.8 ± 8.0	0.3561
Sex	5 F/4 M	7 F/9 M	
Race	8 C/1 AS	12 C/2 AA/1 AS/1 H	
BMI (kg/m^2^)	29.1 ± 4.8	24.7 ± 3.1	0.0103
Minutes of exercise/week	N/A	379.4 ± 175.4	N/A
VO_2_peak (mL/kg/min)	23.5 ± 3.2	40.3 ± 12.1	<0.0001
Isometric Knee Avg Peak Torque (Nm)	187.2 ± 71.0	243.0 ± 92.9	0.1325

*Note*: Data are presented as mean ± SD.

Abbreviations: AA, African American; AS, Asian; C, Caucasian; F, female; H, Hispanic; M, male.

### 3.5 Habitual exercise results in higher glycogen synthesis rates

**FIGURE 3:**
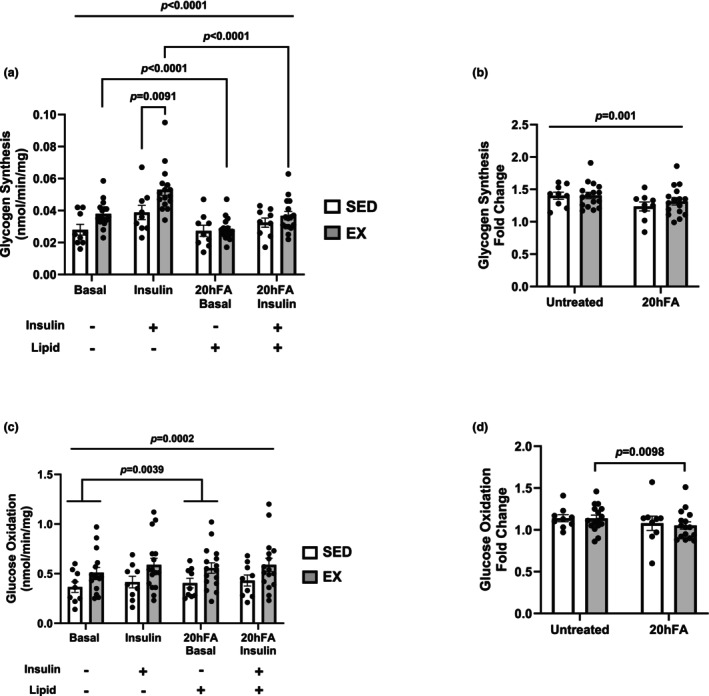
Insulin‐stimulated glycogen synthesis and glucose oxidation rates in highly‐active exercisers (EX) and sedentary (SED) participants. (a) Absolute rates of glycogen synthesis in both basal and insulin‐stimulated and standard and palmitate treated (20hFA) conditions. (b) Relative (fold change over basal) glycogen synthesis in the standard and 20hFA condition. (c) Absolute rates of glucose oxidation in the standard and 20hFA condition. (d) Relative (fold change over basal) glucose oxidation in the standard and 20hFA condition. A three‐way ANOVA was used to detect significance between groups, insulin, and/or treatment, and a two‐tailed *t*‐test was used to distinguish meaningful differences. There was a main effect of insulin (*p* < 0.001) for glycogen synthesis and glucose oxidation (*p* = 0.0002). There was an interaction effect (*p* = 0.0022) with insulin and 20hFA treatment for glycogen synthesis but not glucose oxidation. 20hFA treatment had a main effect for glycogen synthesis fold change (*p* = 0.001) and only in the basal state for absolute values of glucose oxidation (*p* = 0.0039). Data are presented as mean ± SEM. *N* = 9 for SED and *n* = 16 for EX. SED, sedentary participant; EX, highly‐active exerciser; 20hFA, 20‐h 450 μM palmitate treatment.

Insulin incubation significantly increased glycogen synthesis in SED and EX in both the standard and 20hFA conditions (*p* < 0.0001) (Figure 3a). Insulin‐stimulated glycogen synthesis was 37% higher in EX (*p* = 0.0091) while basal rates tended to be elevated (*p* = 0.09) compared to SED (Figure 3a). For EX only, glycogen synthesis rates were blunted by 20hFA treatment (*p* < 0.0001)–basal rates were 34% lower, and insulin rates were 44% lower with 20hFA (Figure 3a). A difference between groups was not detected in the 20hFA condition (Figure 3a). A difference between groups was also not detected in the relative increase in glycogen synthesis with insulin, but there was a main effect of 20hFA treatment (*p* = 0.001) (Figure 3b) resulting in lower fold change values. Insulin incubation had a main effect on glucose oxidation (*p* = 0.0002), and 20hFA treatment affected basal but not insulin‐stimulated oxidation rates (*p* = 0.0039) (Figure 3c). No differences were detected between groups for absolute rates of glucose oxidation (Figure 3c). EX showed a 7% lower relative increase in glucose oxidation with 20hFA treatment (*p* = 0.0098), but SED and EX did not differ in either the standard or 20hFA conditions (Figure 3d).

### 3.6 Insulin signal transduction for SED and EX

**FIGURE 4:**
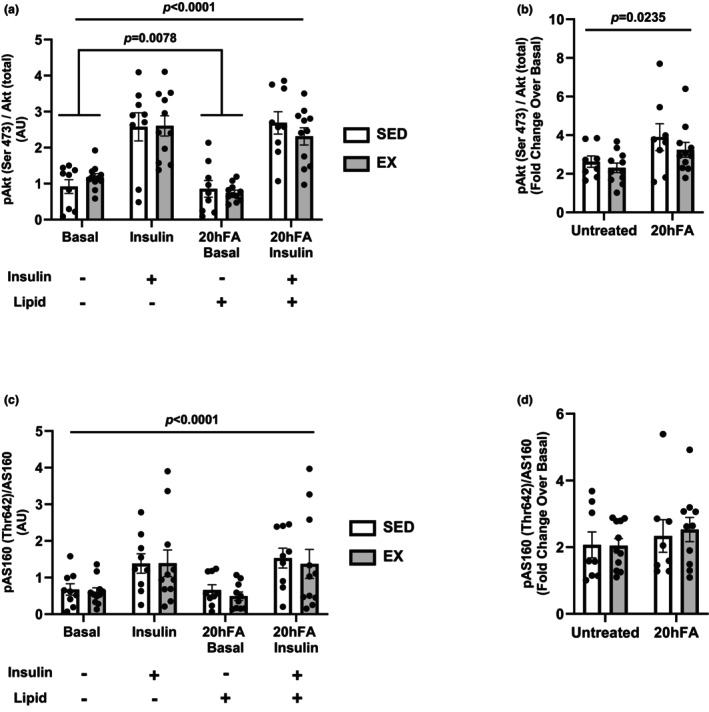
SED and EX insulin signal transduction in the absence or presence of 100 nM insulin and/or 20 h of lipid incubation. (a) Akt (Ser^473^) phosphorylation. (b) Relative (fold change over basal) Akt (Ser^473^) phosphorylation. (c) AS160 (Thr^642^) phosphorylation. (d) Relative (fold change over basal) AS160 (Thr^642^) phosphorylation. A three‐way ANOVA was used to detect significance between groups, insulin, and/or treatment, and a two‐tailed *t*‐test was used to distinguish meaningful differences. There was a main effect of insulin (*p* < 0.0001) for Akt phosphorylation, but 20hFA treatment resulted in an effect in the basal state only (*p* = 0.0078). For AS160, there was a main effect of insulin (*p* < 0.0001) but not 20hFA (*p* > 0.05). 20hFA had a significant effect in only Akt fold change (*p* = 0.0235). There was no group effect for either Akt or AS160 (*p* > 0.05). Data are presented as mean ± SEM. *N* = 9 for SED and *n* = 16 for EX. SED, sedentary participant; EX, highly active exerciser; 20hFA, 20‐h 450 μM palmitate treatment.

There was a significant increase in Akt (Ser^473^) and AS160 (Thr^642^) phosphorylation upon insulin stimulation in the two groups (*p* < 0.0001) in the standard and 20hFA conditions (Figure 4a,c). There was a 20hFA lipid effect for basal phosphorylation of Akt only (*p* = 0.0078) (Figure 4a). No differences between SED and EX were detected in any conditions for either Akt or AS160. 20hFA treatment showed a main effect for the relative increase in Akt phosphorylation with insulin (*p* = 0.0235) (Figure 4b) resulting in higher fold change values, but this was not evident for AS160 (Figure 4d).

## 4 DISCUSSION

Exercise is an effective means of improving insulin action in skeletal muscle, yet the impact of different exercise modalities on insulin action, and how these actions may be imprinted on HSkMCs, remains unclear. Given the evidence to support the metabolic imprinting of exercise training on skeletal muscle (Ceccarelli et al., 2017; Chriett et al., 2017; ACSM, 2024; Tokarz et al., 2023; Vega et al., 2020), in this study we chose to investigate the impact of EE or RE training on insulin action in primary skeletal muscle myotubes derived from human donors. We further employed a method of immunopurification (CD56^+^) of these cells to explore HSkMC‐specific mechanisms, devoid of non‐muscle components. Our main finding was that endurance and resistance training appear to offer similar outcomes in terms of indices of insulin action (glycogen synthesis, glucose oxidation, insulin signal transduction) and no differential protection against the insulin resistance induced by fatty acid treatment (Figure 1). However, when compared to sedentary individuals, HSkMCs derived from habitual exercisers displayed enhanced insulin sensitivity (elevated insulin‐stimulated glycogen synthesis) but without additional protection from fatty acid‐induced insulin resistance (Figure 3).

While both resistance and endurance‐oriented exercise training have been reported to enhance whole‐body insulin action, the cellular mechanisms involved, specifically in skeletal muscle, can vary (Consitt et al., 2019). Differing biological adaptations would seem logical as the microenvironment in skeletal muscle subjected to endurance versus resistance exercise is different. For example, substrate utilization differs between resistance and endurance exercise, evident by increased circulating fatty acids post‐endurance exercise and increased plasma lactate post‐resistance exercise (Blazev et al., 2022). Further, endurance exercise results in greater mitochondrial biogenesis, while resistance exercise results in greater synthesis of contractile proteins (Wilkinson et al., 2008). An important distinction between endurance and resistance exercise training is muscle damage resulting from eccentric contractions, which has been shown to induce insulin resistance (Asp et al., 1996; Asp & Richter, 1996). Insulin resistance from muscle damage is associated with decreases in GLUT4 content and gene transcription, as well as impairments in insulin signaling (Asp, Daugaard, & Richter, 1995; Asp, Kristiansen, & Richter, 1995; Del Aguila et al., 2000; Kristiansen et al., 1997). Additionally, both Akt activity and Ser^473^ phosphorylation can be decreased with eccentric exercise‐induced muscle damage up to 24 h after activity (Del Aguila et al., 2000). Ferrara et al. directly compared the effects of endurance and resistance exercise in older men with overweight and obesity on glucose metabolism using a 6‐month exercise intervention. Exercise was performed 3 days/week; EE was performed at 75%–80% VO_2_peak for 45–60 min/session as treadmill walking/running, and RE was performed at 80% of 1‐repetition maximum for 8–12 repetitions with either one set (for upper body) or two sets (for lower body). The investigators observed that endurance exercise resulted in greater fractional activity of glycogen synthase when activated by insulin (Ferrara et al., 2006). In the present study, muscle biopsies were taken 48 h after the last exercise bout to minimize the acute effects of exercise. We detected no differences between EE and RE for glycogen synthesis or insulin‐mediated phosphorylation of either Akt at Ser^473^ or AS160 at Thr^640^. This suggests that habitual resistance exercise‐associated muscle damage does not result in disproportionate impairments in insulin signaling, and/or the effects of muscle damage may not translate to HSkMCs.

Imprinting of insulin action from in vivo to in vitro models has been previously demonstrated (Snijders et al., 2015). However, the beneficial evidence of exercise training (<6 months) on HSkMCs is debatable, with data suggesting the need for a longer and more consistent exercise stimulus (Goodpaster et al., 2003; Lund et al., 2017; Sigal et al., 2007; Yokoyama et al., 2008). As such, our study incorporated habitual exercisers to explore the impact that long‐term exercise training may have on insulin action in HSkMC. Interestingly, no differences were detected between EE and RE in any measure of insulin action or insulin signaling. Therefore, the participants were combined into an EX group to compare the effects of habitual exercise training to sedentary participants. Although the EX group had higher rates of insulin‐stimulated glycogen synthesis, this metabolic advantage was not preserved when cells were exposed to FA treatment. Multiple studies have shown that exercise‐training‐induced improvements in glucose metabolism are primarily due to increased non‐oxidative glucose disposal (Eriksson et al., 1998; Goodpaster et al., 2003; Yokoyama et al., 2008). Though these studies have largely been completed in populations of individuals with obesity and type 2 diabetes, the present data suggest that this may also occur in lean, healthy individuals. While insulin‐stimulated glycogen synthesis rates were higher in EX, glucose oxidation was similar across all conditions. These results suggest that habitual exercise largely provides improvements in non‐oxidative glucose metabolism, which is in agreement with previous findings (Del Aguila et al., 2000; Ferrara et al., 2006; Kristiansen et al., 1997).

Previously, we and others have shown that incubation of HSkMC with palmitate results in depression of insulin signaling and insulin‐stimulated glycogen synthesis (Bikman et al., 2010; Hage Hassan et al., 2012; Skrobuk et al., 2012). In the present study, we did not observe a palmitate effect consistent with these previous findings in either Akt or AS160 phosphorylation. In our Akt phosphorylation data, 20hFA treatment resulted in a depression of basal values, while insulin‐stimulated values remained unchanged. This resulted in an increase in fold change, which we interpret as an artifact of fold change calculations rather than a biologically meaningful trend for insulin signaling. Notably, a similar pattern was not observed in our AS160 phosphorylation data, where 20hFA treatment resulted in comparable basal and insulin‐stimulated values. Pehmoller et al. also did not see a lipid effect on AS160 Thr^640^ or Akt Ser^473^ phosphorylation when implementing a post‐exercise lipid challenge (Pehmøller et al., 2012). Combined with evidence from other studies that saw no effect of palmitate on insulin signaling and glycogen synthesis in myotubes (Chriett et al., 2017; Tokarz et al., 2023), this may suggest that palmitate has other mechanisms of action. We suggest that future studies investigate fatty acid oxidation and storage to better understand these differences in the effects of palmitate treatment.

A strength of the current study was the utilization of a well‐trained population of habitual exercisers, with subjects participating in exercise well above current American College of Sports Medicine guidelines of 150 min per week (ACSM, 2024). No differences were detected for myoblast expansion time or myotube protein content (data not shown), signifying the improvements in insulin action occurred independently from changes in HSkMC cell growth and differentiation with exercise training. One limitation of this study is the small number of subjects per group analyzed for the EE versus RE insulin signaling data (*n* = 5–6), which reduced statistical power to detect subtle group differences. Nonetheless, the Akt and AS160 phosphorylation data align with our glycogen synthesis and glucose oxidation data and provide a consistent narrative. Limitations of the present study also include the lack of dietary control with our participants. Although all participants were habitually active and otherwise healthy, macronutrient intake or caloric intake was not controlled for in this study. Additionally, it is important to note that all experiments were done in HSkMCs. While HSkMCs retain donor characteristics and allow for mechanistic insight, they do not capture the complexity of whole‐body physiology, including endocrine signaling. As such, these findings are reflective of intrinsic properties of muscle rather than whole‐body metabolic characteristics. Future studies implementing dietary standardization and in vivo measurements are warranted to build on the current study's findings and better understand the interaction between exercise modality and skeletal muscle insulin action.

In conclusion, in the present study no differences were detected in HSkMCs from habitual endurance and resistance exercisers in basal or insulin‐stimulated glycogen synthesis, glucose oxidation, or phosphorylation of Akt and AS160. Additionally, neither exercise mode appeared to provide protection against the insulin resistance induced by fatty acid exposure. Compared to sedentary individuals, HSkMCs of habitual exercisers demonstrated enhanced insulin‐stimulated glycogen synthesis; however, habitual exercise did not appear to provide intrinsic protection against fatty acid‐induced insulin resistance. Together, these findings suggest that habitual exercise can result in innate changes to some aspects of insulin action, regardless of exercise mode.

## FUNDING INFORMATION

Clin Trial #NCT04334343, Grant #RO1 DK120322 (to JAH, LS), Grant #U01 AR071128 (to JAH, WEK).

## CONFLICT OF INTEREST STATEMENT

The authors declare no conflicts of interest, financial or otherwise.

## Supporting information


**Figure S1.** Representative images for Akt and AS160 blots.

## Data Availability

Data will be made available upon reasonable request.
