# Supplementary figures and images for: Effects of habitual endurance and resistance exercise on insulin action in primary human skeletal muscle stem cells
Source: Physiol Rep. 2025 Sep 30;13(19):e70600. doi: 10.14814/phy2.70600 (PMC12484306; doi:10.14814/phy2.70600)

**Supplemental Materials**

**
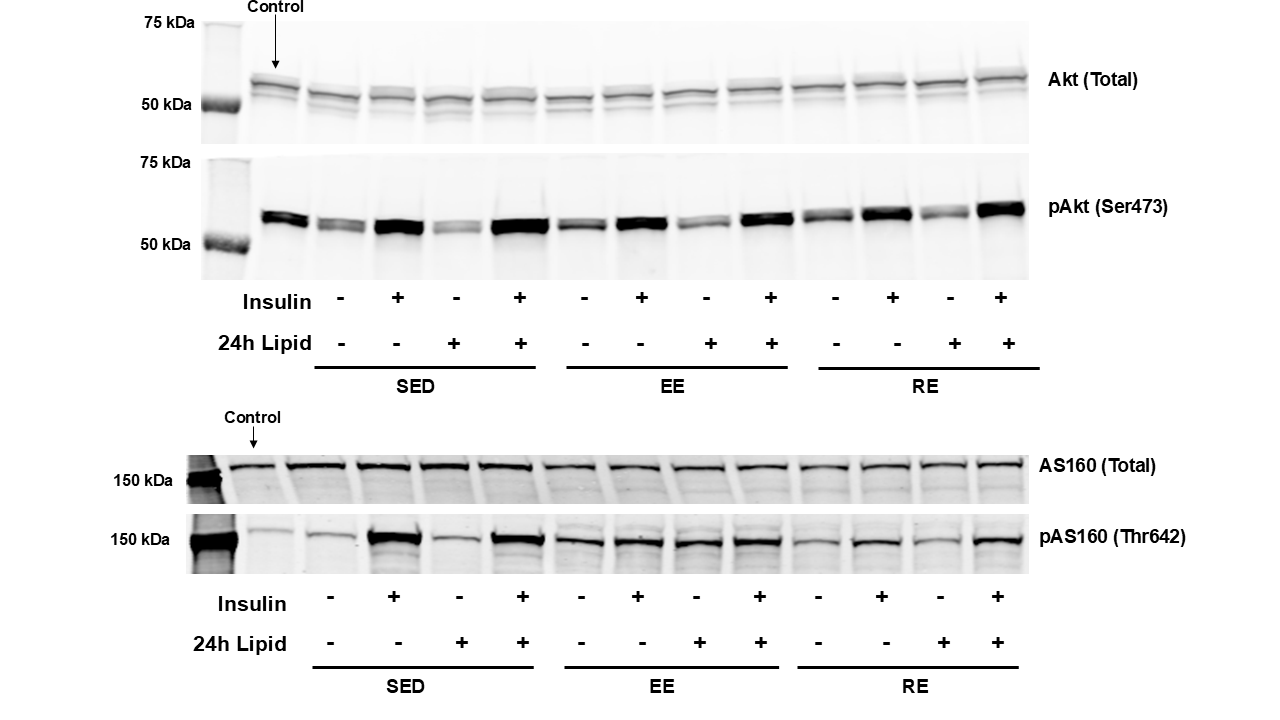
**

Supplemental Figure 1. Representative images for Akt and AS160 blots.

Supplement: Supplementary file 1 — Figure S1. Representative images for Akt and AS160 blots. [file PHY2-13-e70600-s001.docx]
